# Mental Health-Related Work Disabilities During the COVID-19 Lockdown in Spain: A Retrospective Analysis

**DOI:** 10.3390/healthcare13030236

**Published:** 2025-01-24

**Authors:** Eva María Gutiérrez Naharro, José Fernández Sáez, Amalia Sillero Sillero, Pau Tolo Espinet, José Antonio Ponce Blandón

**Affiliations:** 1 Escola Universitària Gimbernat, Adscrita a Universitat Autònoma de Barcelona, 08174 Sant Cugat, Spain; eva.gutierrez@eug.es; 2 Nursing Department, Faculty of Nursing, Physiotherapy and Podiatry, University of Seville, 41009 Seville, Spain; japonce@us.es; 3 Facultad de Enfermería, Campus Terres de l’Ebre, Universitat Rovira i Virgili, 43500 Tarragona, Spain; 4 Servei Atenció Primaria Terres de l’Ebre, Institut Català de la Salut, 43500 Tarragona, Spain; 5 Escuela de Ciéncias de la Salud, Universidad de VIC-Central (UVIC-UCC), 08242 Manresa, Spain; ptolo@umanresa.cat; 6 Mutua Intercomarcal, 08242 Manresa, Spain

**Keywords:** mental health-related work disability, COVID-19 lockdown, public health crisis, Mutual Collaborator with Social Security, psychological impact, pandemic-related anxiety

## Abstract

**Background:** The COVID-19 lockdown posed unprecedented psychological challenges worldwide. In Spain, Mutual Collaborators with Social Security manage work-related disabilities, including mental health cases. **Objectives:** To describe and analyze work-related disabilities with mental health diagnoses during the COVID-19 lockdown in Spain from the perspective of a Mutual Collaborator with Social Security in the Spanish healthcare system. **Methods:** Descriptive, retrospective, and cross-sectional study of a sample of 5135 patients. Descriptive statistics reported mean values and standard deviation by sex and age. Inferential analyses were conducted using the Mann–Whitney U-test and correlation analysis. **Results:** The study population included 5135 patients managed by a Mutual Collaborator with Social Security during the COVID-19 lockdown, 63.5% of whom were women. Cantabria reported the highest average sick leave duration (62.80 days), while La Rioja had the lowest (39.19 days). Generalized anxiety disorder was the most prevalent diagnosis (69.17%), followed by adaptive disorders and mild depression. Women had a slightly higher prevalence of anxiety, while men showed higher rates of adaptive disorders. **Conclusions:** The findings underscore the psychological impact of the COVID-19 lockdown, revealing significant sex and regional differences in mental health diagnoses and sick leave duration. Generalized anxiety disorder was the predominant diagnosis, highlighting the need for targeted mental health interventions during crises.

## 1. Introduction

The COVID-19 pandemic created an unprecedented global crisis with significant consequences worldwide [1]. The public health emergency caused by this novel disease required quarantine measures in many countries and the isolation of infected individuals to reduce the risk of contagion [2,3]. People exposed to the stress of the outbreak may experience marked distress, leading to adjustment disorders, and in cases of persistent sadness, major depressive disorder (MDD) can develop [4].

Depression can manifest and worsen due to multiple factors, such as genetic predispositions, personal experiences, and social-environmental conditions [5]. In the context of the COVID-19 pandemic, various studies have reported a significant increase in stress and anxiety levels, which can lead to the onset or exacerbation of depressive symptoms [6,7]. Moreover, the coexistence of multiple mental disorders often shares common etiological pathways, further complicating clinical management [8].

In Spain, psychiatric disorders are the second leading cause of temporary disability [9]. Globally, psychiatric conditions account for approximately 25% of the disease burden, as reported by the World Bank and the World Health Organization (WHO), resulting in substantial economic repercussions [10]. The mental health toll of quarantine measures is evident, with large-scale studies underway to better understand the effects of lockdowns, particularly in Europe. During pandemics, fear increases stress and anxiety in healthy individuals while exacerbating symptoms in those with pre-existing mental health disorders [11]. These conditions can evolve into depression, panic attacks, PTSD, psychotic symptoms, and even suicide [12], especially in quarantined patients, who tend to experience higher levels of psychological stress [2,7].

Mutual Collaborators with Social Security (MCSS)—formerly known as Mutual Societies for Work Accidents and Occupational Diseases until 2014—are private, non-profit organizations operating under the supervision of the Ministry of Labor and Social Economy. Their primary role is to manage professional contingencies, including healthcare services. They may also oversee the management of temporary disability benefits for common contingencies (ITCC) and provide prevention activities for affiliated companies [13].

A “common contingency” (CC) is defined as a situation in which a worker is unable to perform their job due to a non-work-related illness or injury lasting more than 72 h, receiving healthcare through the Public Health System (SPS) [14]. Psychiatric disorders are highly prevalent among primary care patients, with studies estimating a prevalence rate of 23–30% in the general population [9]. Specifically, these conditions disproportionately affect women, younger individuals, and those with lower educational and cultural levels. Women are more likely to experience generalized anxiety disorder and depression, while younger individuals often present with adjustment disorders [15]. Additionally, lower educational and cultural levels are associated with reduced access to mental health resources and delayed treatment initiation, exacerbating the chronicity and severity of these conditions [16]. Psychiatric disorders are typically chronic, prone to relapses, and often necessitate prolonged periods of temporary disability, occasionally progressing to permanent incapacity [17].

Emerging mental health issues associated with SARS-CoV-2 may lead to long-term health challenges. Evidence from past epidemics, such as the 2003 SARS-CoV outbreak and the 2012 MERS-CoV outbreak, revealed that up to 35% of SARS survivors developed psychiatric symptoms during recovery [18,19], while nearly 40% of MERS patients required psychiatric intervention [20]. These historical precedents underscore the urgency of understanding the mental health impacts of COVID-19 to inform public health strategies and interventions better.

For this reason, this study aims to describe and analyze mental health-related temporary disabilities during the COVID-19 lockdown in Spain, with a focus on their prevalence, duration, and demographic characteristics, including sex, age, and regional distribution. These disabilities include Generalized Anxiety Disorder (GAD), adjustment disorder with Conduct Disturbance, Major Depressive Disorder (Single Mild Episode), Nervousness, Acute Stress Reaction, Neurotic Depression, Unspecified Depression, Panic Disorder (Episodic Paroxysmal Anxiety), Major Depressive Disorder (Recurrent), Agoraphobia (with/without Panic Attacks), Social Phobias, Demoralization and Apathy, and Unspecified Insomnia.

## 2. Materials and Methods

### 2.1. Design and Setting

This study employed a descriptive, retrospective, observational, cross-sectional, and analytical design. It aimed to assess the prevalence of temporary incapacity for work due to common contingencies (ITCC) with a diagnosis of mental health conditions during the COVID-19 lockdown period from 14 March 2020 to 21 June 2020 among workers affiliated with the Asepeyo mutual insurance, Spain. The study utilized pre-existing data from medical records and databases, focusing on events and variables recorded before and during the specified period. The study was conducted in Spain, and the analysis included all patients who experienced ITCC due to mental health conditions during the specified period.

### 2.2. Sample

The sample consisted of patients who met the following inclusion criteria. (1) Individuals who ITCC leave certified by the Public Health Service (SPS) with a mental health diagnosis. (2) Patients whose incapacity was managed by Asepeyo mutual insurance during the COVID-19 lockdown period. The exclusion criteria were as follows. (1) Patients who were already on psychiatric leave before the COVID-19 lockdown. (2) Patients with relevant mental health diagnoses who have not been evaluated by the responsible healthcare personnel. (3) Cases where psychiatric conditions are deemed unrelated to COVID-19. (4) Patients whose medical records lacked sufficient data to confirm the mental health diagnosis or the duration of ITCC leave.

### 2.3. Procedure

Data for this study were exclusively obtained from the *CHAMAN* database, maintained by Asepeyo, and authorized by Social Security in compliance with the applicable Spanish data protection regulations (LOPD). Patient records were pseudo-anonymized using numerical codes (Medical Record Number) and based on ICD-10 coded diagnoses. No personally identifiable information, such as names, surnames, or ID numbers, was accessed.

### 2.4. Variables

The primary variables of interest in this study were the prevalence of ITCC due to mental health conditions, defined as the frequency of patients diagnosed with mental health disorders who were granted ITCC during the study period, and the duration of ITCC, measured as the total number of days on leave for each patient. Secondary variables included sex, analyzed to explore potential differences in ITCC prevalence between genders and age and examined to assess correlations between age and the duration of incapacity.

### 2.5. Data Collection

Data were collected from the *CHAMAN* database, a secure database managed by Asepeyo mutual insurance. The records were accessed under strict data protection protocols to ensure patient confidentiality. Data were pseudo-anonymized to maintain the anonymity of participants. The database contains records based on ICD-10 diagnosis codes and is authorized by Social Security for use in research. Only data relevant to the study, such as ITCC records, diagnosis, age, and sex, were extracted.

### 2.6. Statistical Analysis

Descriptive analysis was conducted using frequencies and percentages for categorical variables (e.g., sex) and means with standard deviations for continuous variables (e.g., age and total days on leave). The analysis was carried out for the total sample and stratified by ACs. A Z-test for differences in proportions was applied to prevalence data to assess differences by sex across ACs. The nonparametric Mann–Whitney U-test was used to compare age and days on leave between different sex groups.

Statistical analyses were performed using SPSS version 26.0 (IBM, Armonk, NY, USA), with a significance level set at *p* < 0.05.

### 2.7. Ethical Considerations

This study was approved by the Asepeyo Ethical Committee, Spain (Approval Code: 2022/48-MLA-ASEPEYO. Approval Date: 31 May 2022). All procedures adhered to the ethical principles of the Declaration of Helsinki (October 2013, Fortaleza, Brazil). This study ensured strict confidentiality of patient data, and no personally identifiable information was used.

## 3. Results

The Asepeyo mutual insurance population who experienced temporary incapacity for work (ITCC) with a mental health-related diagnosis during the COVID-19 lockdown period in 2020 consisted of 5135 patients, with 3259 (63.5%) being women and 1876 (36.5%) men. Across all Autonomous Communities (ACs.), more women than men experienced ITCC; however, this difference was not statistically significant in Murcia and La Rioja. The regions with the highest proportion of women experiencing ITCC were Aragón (83.8%), followed by Castilla-La Mancha (70.9%) and Castilla y León (69.7%). The overall mean age was 44.4 years, with a standard deviation of 0.49 (Table 1).

**Figure 1:**
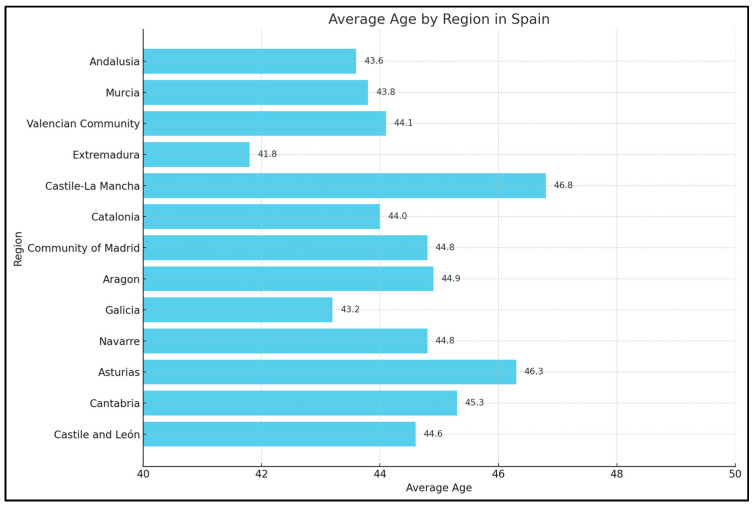
Average Disability Days by Region and Gender.

Castilla-La Mancha had the highest mean age (46.8 years), with women having a mean age of 47.7 years and men 44.5 years. In contrast, Extremadura had the lowest mean age at 41.8 years, with women having a mean age of 42.8 years and men 39.7 years. Other regions with lower average ages included Illes Balears (43.0 years), Andalucía (43.6 years), and Canarias (43.8 years). The only statistically significant difference in mean age between men and women was observed in Andalucía (*p* = 0.001). Table 2 and Figure 1.

**Figure 2:**
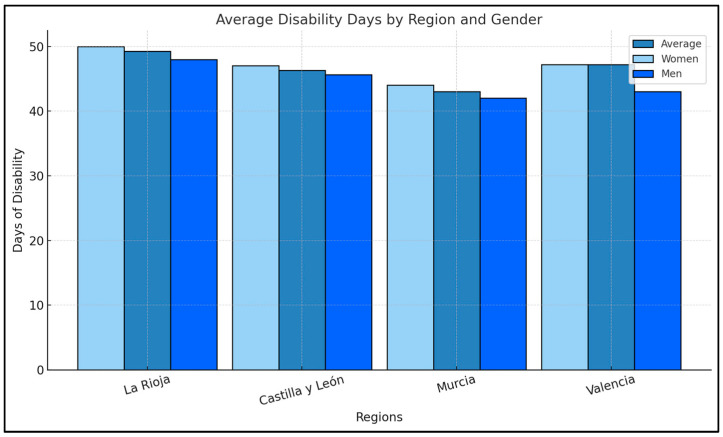
Average Age by Region and Gender in Spain.

Cantabria had the highest average number of sick leave days, with a mean of 62.80 days, while La Rioja had the lowest, 39.19 days. Significant differences in the average number of sick leave days were observed in Castilla y León (*p* = 0.002). Table 3 and Figure 2.

The most prevalent diagnoses identified in this study were 13, ranked from the highest to the lowest prevalence: Generalized anxiety disorder (69.17%), with 2252 women (69.10%) and 1300 men (69.30%). (1) Adjustment disorder with conduct disturbance (11.47%), with 351 women (10.77%) and 238 men (12.69%). (2) Major depressive disorder, single mild episode (7.52%), with 236 women (7.24%) and 150 men (8%). (3) Nervousness (3.14%), with 100 women (3.07%) and 61 men (3.25%). (4) Acute stress reaction (2.59%), with 102 women (3.13%) and 31 men (1.65%), statistically significant (*p* < 0.001) (Table 4).

## 4. Discussion

This study sheds light on the significant impact of the COVID-19 pandemic on the mental health of workers, as evidenced by the analysis of 5135 cases of temporary incapacity for work due to mental health disorders managed by *MCSS Asepeyo*.

The findings align with previous research that has highlighted the extensive mental health repercussions of global health crises, such as SARS and MERS, where the combination of isolation and uncertainty amplifies psychological distress [18,19]. Generalized anxiety disorder (GAD) emerged as the most frequently diagnosed condition, accounting for 69.17% of cases, followed by adjustment disorders (11.47%) and mild major depressive disorder (7.52%). These trends are consistent with global patterns observed during the pandemic [6,15].

One of the most important findings is the gender disparity in ITCC cases, with women comprising 63.5% of the cohort. This trend is consistent with studies, such as by Hartman et al. [21], which indicate that women are disproportionately affected by mental health conditions due to biological predispositions, societal expectations, and occupational pressures, including caregiving responsibilities and work environments with reduced job security The additional caregiving and professional demands that many women faced during the pandemic likely compounded their mental health challenges [22]. This gender-specific vulnerability is further underscored by the higher prevalence of acute stress reactions in women (3.13%) compared to men (1.65%). Similar patterns were observed by Pappa et al. [15], who documented elevated rates of anxiety, depression, and insomnia among women, particularly those working in healthcare, during the pandemic.

The age group most affected by the pandemic’s mental health impacts consisted of individuals aged 40–49, with an average age of 44.4 years in the studied cohort. This observation aligns with findings by Charlson et al. [23], who reported a peak prevalence of anxiety disorders in this demographic. The dual pressures of professional responsibilities and family obligations likely contribute to the heightened vulnerability of middle-aged adults. These results highlight the necessity of tailored mental health interventions for this age group.

Regional disparities also provide significant insights into the pandemic’s mental health impact. While the study focused on various regions, evidence from Andalusia, as highlighted in Rodríguez-Rey et al., [24] indicates elevated levels of psychological distress in the general population during the initial stages of the pandemic [2]. Women in Andalusia were particularly vulnerable, facing unique challenges stemming from professional caregiving roles and family obligations. This aligns with broader findings from our cohort, emphasizing the disproportionate burden borne by women during the pandemic. Addressing these regional-specific dynamics is critical for developing equitable and effective mental health interventions.

Cantabria recorded the most extended average duration of sick leave (62.80 days), while La Rioja had the shortest (39.19 days). Such regional variations likely reflect differences in healthcare infrastructure, socioeconomic conditions, and the availability of mental health resources. Vicente-Pardo et al. highlighted similar findings during the pandemic, emphasizing the importance of harmonizing mental healthcare policies and ensuring equitable resource allocation [25].

This study also brings attention to the chronic nature of some mental health conditions, particularly generalized anxiety disorder. Research by Cuijpers et al. [26] has shown that such disorders can persist for years if left untreated, raising questions about the readiness of affected individuals to reintegrate into the workforce. These findings underscore the critical need for sustained therapeutic and occupational support to facilitate long-term recovery and effective reintegration.

### 4.1. Strengths and Limitations

This study provides a robust analysis of ITCC cases during the pandemic, leveraging an extensive and comprehensive dataset to examine trends in diagnostic prevalence, demographic patterns, and regional disparities. Including gender-specific and region-specific analyses offers valuable insights that can inform more tailored mental health policies.

However, this study has certain limitations. The retrospective nature of the design restricts the ability to draw causal inferences. Additionally, relying on data from a single mutual insurance organization, Asepeyo may limit the findings’ generalizability to other regions or populations. The exclusion of individuals already on sick leave for mental health conditions before the pandemic could also result in an underestimation of the actual burden of these disorders.

### 4.2. Implications for Practice and Future Research

The findings emphasize the need for proactive strategies, such as gender-sensitive interventions and tailored support for middle-aged workers, to address the psychological toll of the pandemic. Addressing regional disparities through equitable resource allocation and program stabilization can strengthen workforce resilience for future crises.

Policymakers, healthcare providers, and employers can develop effective strategies to support a healthier, more resilient workforce capable of navigating future public health challenges.

Future investigations should prioritize longitudinal studies to monitor recovery trajectories and assess the effectiveness of mental health interventions implemented during the pandemic. Investigating the particular vulnerabilities of high-stress occupations, such as those in healthcare, could further inform targeted support strategies, as suggested by Pappa et al. and other studies focusing on psychological distress during health crises [15,27]. Additionally, incorporating qualitative perspectives could provide a richer understanding of the lived experiences of affected workers, complementing quantitative findings.

## 5. Conclusions

This study highlights the significant psychological impact of the COVID-19 pandemic on the Spanish workforce, particularly among those with temporary incapacity for work due to mental health diagnoses. Key findings include gender disparities, age-specific vulnerabilities, and regional differences in sick leave duration and prevalence. The results indicated that 63.5% of the patients were especially vulnerable women, possibly due to additional responsibilities during the pandemic. Aragon reported the highest percentage of affected women (83.8%).

The average age of the patients was 44.4 years, with women being older on average (47.7 years) than men (44.5 years). Regarding the duration of sick leave, Cantabria had the highest average duration (62.8 days), with notable differences between men (70.4 days) and women (58.8 days). In contrast, La Rioja recorded the shortest duration (39.19 days).

Generalized anxiety disorder was the most common diagnosis, accounting for 69.17% of cases, followed by adjustment disorders (11.47%) and mild major depressive disorder (7.52%). Women exhibited higher rates of acute stress reactions (3.13%) compared to men.

This analysis reveals how factors such as sex and regional differences influenced emotional responses during the pandemic. These findings underscore the need for mental health policies tailored to gender and regional contexts to enhance care delivery during future crises. By addressing the chronicity of conditions like generalized anxiety disorder and ensuring sustained support, policymakers can foster a healthier and more resilient workforce.

Future research should focus on exploring the long-term implications of the pandemic on mental health and refine intervention strategies to better support workforce well-being.

## Figures and Tables

**Table 1:** Patients with mental health-related ITCC during COVID-19 lockdown, by sex and ACs n = 5135.

ACs	Total (*n*)	Female (*n*, %)	Male (*n*, %)	*p*-Value
Andalucia	561	366 (65.2%)	195 (34.8%)	**<0.001**
Aragon	105	88 (83.8%)	17 (16.2%)	**<0.001**
Asturias	116	71 (61.2%)	45 (38.8%)	**0.001**
Canarias	141	89 (63.1%)	52 (36.9%)	**<0.001**
Cantabria	35	23 (65.7%)	12 (34.3%)	**0.009**
Castilla Leon	267	186 (69.7%)	81 (30.3%)	**<0.001**
Castilla La Mancha	79	56 (70.9%)	23 (29.1%)	**<0.001**
Catalunya	2608	1615 (61.9%)	993 (38.1%)	**<0.001**
Valenciana	300	187 (62.3%)	113 (37.7%)	**<0.001**
Euskadi	250	153 (61.2%)	97 (38.8%)	**<0.001**
Extremadura	29	20 (69.0%)	9 (31.0%)	**0.004**
Galicia	183	119 (65.0%)	64 (35.0%)	**<0.001**
Illes Balears	42	29 (69.0%)	13 (31.0%)	**<0.001**
La Rioja	42	24 (57.1%)	18 (42.9%)	0.190
Madrid	213	139 (65.3%)	74 (34.7%)	**<0.001**
Murcia	68	38 (55.9%)	30 (44.1%)	0.170
Navarra	96	56 (58.3%)	40 (41.7%)	**0.021**

Data are shown as frequency (percentage) for categorical variables. Significant differences are highlighted in bold.

**Table 2:** Age and sex distribution of patients with mental-health related ITCC during COVID-19 lockdown, ACs.

ACs	Total Mean (SD)	Female Mean (SD)	Male Mean (SD)	*p*-Value
Andalucia	43.6 (9.8)	42.5 (9.6)	45.5 (9.9)	<0.001 ***
Aragon	44.9 (10.2)	44.2 (10.2)	48.7 (9.1)	0.101
Asturias	46.3 (10.7)	44.9 (11.1)	48.6 (9.5)	0.070
Canarias	43.8 (9.7)	42.5 (10.5)	46.0 (7.8)	0.033 *
Cantabria	44.7 (10.4)	42.9 (9.1)	48.2 (12.2)	0.208
Castilla Leon	44.6 (11.1)	45.2 (10.9)	43.4 (11.3)	0.211
Castilla-Mancha	46.8 (11.1)	47.7 (10.9)	44.5 (11.4)	0.257
Catalunya	44.0 (10.8)	44.0 (10.9)	44.1 (10.7)	0.757
Valenciana	44.1 (10.0)	44.0 (10.0)	44.1 (9.9)	0.869
Pais Vasco	45.3 (9.6)	44.9 (9.7)	45.9 (9.4)	0.435
Extremadura	41.8 (11.0)	42.8 (10.8)	39.7 (11.8)	0.523
Galicia	43.2 (10.3)	42.2 (9.7)	45.1 (11.2)	0.036 *
Illes Balears	43.0 (10.9)	42.7 (10.9)	43.6 (11.3)	0.936
La Rioja	44.9 (10.0)	42.4 (10.7)	48.3 (8.2)	0.056
Madrid	44.8 (9.8)	44.8 (10.9)	44.8 (8.9)	0.892
Murcia	44.0 (10.4)	42.8 (9.6)	45.5 (11.3)	0.269
Navarra	44.8 (10.4)	44.4 (11.5)	45.4 (8.5)	0.696

Data are shown as mean (standard deviation). Significance is shown as * *p* < 0.05 and *** *p* < 0.001.

**Table 3:** Sick leave duration of patients with mental health-related ITCC during COVID-19 lockdown, by ACs.

ACs	Total Mean (SD)	Female Mean (SD)	Male Mean (SD)	*p*-Value
Andalucia	39.70 (28.08)	40.2 (28.0)	38.8 (28.3)	0.501
Aragon	55.03 (27.70)	54.8 (27.7)	56.2 (28.5)	0.787
Asturias	44.47 (29.69)	44.8 (29.6)	43.9 (30.2)	0.841
Canarias	51.55 (27.98)	48.9 (26.2)	56.1(30.5)	0.219
Cantabria	62.80 (27.69)	58.8 (28.7)	70.4 (25.0)	0.465
Castilla—Leon	48.36 (29.40)	52.1 (29.4)	39.8 (27.7)	0.002 *
Castilla-La Mancha	46.33 (29.66)	49.8 (28.8)	38.0 (30.8)	0.070
Catalunya	45.53 (28.58)	45.4 (28.7)	45.7 (28.4)	0.843
Valenciana	45.66 (25.69)	47.2 (25.3)	43.0 (26.2)	0.109
Pais Vasco	45.88 (28.13)	43.8 (27.5)	49.2 (28.9)	0.148
Extremadura	44.59 (28.05)	51.3 (28.9)	29.8 (20.4)	0.094
Galicia	52.73 (27.73)	54.9 (27.4)	48.7 (28.0)	0.149
Illes Balears	46.29 (28.05)	46.3 (30.1)	46.2 (24.1)	0.809
Madrid	49.27 (26.23)	49.9 (26.5)	48.1 (25.8)	0.666
Murcia	51.69 (25.89)	52.8 (24.2)	50.3 (28.3)	0.800
Navarra	42.02 (30.85)	44.1(31.4)	39.1 (30.1)	0.572

Data are shown as mean (standard deviation). Significance is shown as * *p* < 0.05.

**Table 4:** The most prevalent diagnoses during the COVID-19 lockdown across ACs.

Diagnosis	Total (*n*, %)	Female (*n*, %)	Male (*n*, %)	*p*-Value
Generalized Anxiety Disorder	3552 (69.17%)	2252 (69.10%)	1300 (69.30%)	0.884
Adjustment Disorder with Conduct Disturbance	589 (11.47%)	351 (10.77%)	238 (12.69%)	0.038 *
Major Depressive Disorder, Single Mild Episode	386 (7.52%)	236 (7.24%)	150 (8.00%)	0.324
Nervousness	161 (3.14%)	100 (3.07%)	61 (3.25%)	0.717
Acute Stress Reaction	133 (2.59%)	102 (3.13%)	31 (1.65%)	0.001 *
Neurotic Depression	111 (2.16%)	83 (2.55%)	28 (1.49%)	0.012 *
Unspecified Depression	74 (1.44%)	53 (1.63%)	21 (1.12%)	0.142
Panic Disorder (Episodic Paroxysmal Anxiety)	49 (0.95%)	34 (1.04%)	15 (0.80%)	0.387
Major Depressive Disorder, Recurrent	48 (0.93%)	26 (0.80%)	22 (1.17%)	0.179
Agoraphobia with/without Panic Attacks	8 (0.16%)	7 (0.21%)	1 (0.05%)	0.158
Social Phobias	12 (0.23%)	9 (0.28%)	3 (0.16%)	0.406
Demoralization and Apathy	5 (0.10%)	3 (0.09%)	2 (0.11%)	0.872
Unspecified Insomnia	7 (0.14%)	3 (0.09%)	4 (0.21%)	0.257

Data are shown as frequency (percentage) for categorical variables. Significance is shown as * *p* < 0.05.

## Data Availability

Aggregated data supporting this study’s findings are available upon reasonable request from the corresponding author, A.S.-S., and are subject to review. These data are not publicly available due to privacy concerns and the potential for compromising research participant privacy/consent.

## References

[1] Shigemura J., Ursano R.J., Morganstein J.C., Kurosawa M., Benedek D.M. (2020). Public responses to the novel 2019 coronavirus (2019-nCoV) in Japan: Mental health consequences and target populations. Psychiatry Clin. Neurosci..

[2] Brooks S.K., Webster R.K., Smith L.E., Woodland L., Wessely S., Greenberg N., Rubin G.J. (2020). The psychological impact of quarantine and how to reduce it: Rapid review of the evidence. Lancet.

[3] Nussbaumer-Streit B., Mayr V., Dobrescu A.I., Chapman A., Persad E., Klerings I., Wagner G., Siebert U., Ledinger D., Zachariah C. (2020). Quarantine alone or in combination with other public health measures to control COVID-19: A rapid review. Cochrane Database Syst. Rev..

[4] Huremovic D. (2019). Psychiatry of Pandemics: A Mental Health Response to Infection Outbreak.

[5] Kupfer D.J., Frank E., Phillips M.L. (2012). Major depressive disorder: New clinical, neurobiological, and treatment perspectives. Lancet.

[6] Wickens C.M., Popal V., Fecteau V., Amoroso C., Stoduto G., Rodak T., Li L.Y., Hartford A., Wells S., Elton-Marshall T. (2023). The mental health impacts of the COVID-19 pandemic among individuals with depressive, anxiety, and stressor-related disorders: A scoping review. PLoS ONE.

[7] Styra R., Hawryluck L., Robinson S., Kasapinovic S., Fones C., Gold W.L. (2008). Impact on healthcare workers employed in high-risk areas during the Toronto SARS outbreak. J. Psychosom. Res..

[8] Hill J.-E., Harris C., Danielle L.-C. (2022). The prevalence of mental health conditions in healthcare workers during and after a pandemic: Systematic review and meta-analysis. J. Adv. Nurs..

[9] Henares Montiel J., Ruiz-Pérez I., Sordo L. (2020). Mental Health in Spain and Differences by Gender and Autonomous Communities. Gac. Sanit..

[10] Velavan T.P., Meyer C.G. (2020). The COVID-19 epidemic. Trop. Med. Int. Health.

[11] Xiang Y.T., Yang Y., Li W., Zhang L., Zhang Q., Cheung T., Ng C.H. (2020). Timely mental health care for the 2019 novel coronavirus outbreak is urgently needed. Lancet Psychiatry.

[12] World Health Organization (1946). Constitution of the World Health Organization.

[13] Castellano M., Molina A. (2006). La IT y su control médico. Aspectos médico-legales. La Mutua. Rev. Téc. Salud Lab. Prev..

[14] Royal Decree 625/2014, of July 18, Regulating Certain Aspects of the Management and Control of Temporary Disability Processes During the First 365 Days of Their Duration. B.O.E. 2014, p. 176. https://www.boe.es/diario_boe/txt.php?id=BOE-A-2014-12345.

[15] Pappa S., Ntella V., Giannakas T., Giannakoulis V.G., Papoutsi E., Katsaounou P. (2020). Prevalence of depression, anxiety, and insomnia among healthcare workers during the COVID-19 pandemic: A systematic review and meta-analysis. Brain Behav. Immun..

[16] Losada-Baltar A., Jiménez-Gonzalo L., Gallego-Alberto L., Pedroso-Chaparro M.D.S., Fernandes-Pires J., Márquez-González M. (2020). “We Are Staying at Home”. Association of Self-perceptions of Aging, Personal and Family Resources, and Loneliness With Psychological Distress During the Lock-Down Period of COVID-19. J. Gerontol. Ser. B.

[17] Jiménez J.F., Martínez J.A., Rodado C., Martínez D., Sánchez P., Reyes A. (1996). Incidence of Sick Leaves in an Urban Health Centre: Considerations Regarding the Diagnostic Groups (WONCA). That Orig. Them Med. Trab..

[18] Wu K.K., Chan S.K., Ma T.M. (2005). Posttraumatic stress after SARS. Emerg. Infect. Dis..

[19] Mak I.W., Chu C.M., Pan P.C., Yiu M.G., Chan V.L. (2009). Long-term psychiatric morbidities among SARS survivors. Gen. Hosp. Psychiatry.

[20] Kim H.C., Yoo S.Y., Lee B.H., Lee S.H., Shin H.S. (2018). Psychiatric findings in suspected and confirmed Middle East Respiratory Syndrome patients quarantined in hospital: A retrospective chart analysis. Psychiatry Investig..

[21] Hartman C.A., Larsson H., Vos M., Bellato A., Libutzki B., Solberg B.S., Chen Q., Du Rietz E., Mostert J.C., Kittel-Schneider S. (2023). Anxiety, Mood, and substance use disorders in Adult Men and Women with and without ADHD: A Substantive and Methodological Overview. Neurosci. Biobehav. Rev..

[22] Bezerra H.S., Alves R.M., Nunes A.D.D., Barbosa I.R. (2021). Prevalence and Associated Factors of Common Mental Disorders in Women: A Systematic Review. Public Health Rev..

[23] Charlson F., van Ommeren M., Flaxman A., Cornett J., Whiteford H., Saxena S. (2019). New WHO Prevalence Estimates of Mental Disorders in Conflict Settings: A Systematic Review and Meta-Analysis. Lancet.

[24] Rodríguez-Rey R., Garrido-Hernansaiz H., Collado S. (2020). Psychological Impact and Associated Factors During the Initial Stage of the Coronavirus (COVID-19) Pandemic Among the General Population in Spain. Front. Psychol..

[25] Vicente-Pardo J.M., López-Guillén-García A. (2021). Labor Incapacity during COVID-19, Preventive Aspects, and Consequences. Med. Segur. Trab..

[26] Cuijpers P., Sijbrandij M., Koole S.L., Huibers M.J.H., Berking M., Andersson G. (2014). Psychological Treatment of Generalized Anxiety Disorder: A Meta-Analysis. Clin. Psychol. Rev..

[27] Glowacz F., Schmits E. (2020). Psychological distress during the COVID-19 lockdown: The young adults most at risk. Psychiatry Res..

